# Insulin Signaling, Lifespan and Stress Resistance Are Modulated by Metabotropic GABA Receptors on Insulin Producing Cells in the Brain of *Drosophila*

**DOI:** 10.1371/journal.pone.0015780

**Published:** 2010-12-30

**Authors:** Lina E. Enell, Neval Kapan, Jeannette A. E. Söderberg, Lily Kahsai, Dick R. Nässel

**Affiliations:** Department of Zoology, Stockholm University, Stockholm, Sweden; University of Texas MD Anderson Cancer Center, United States of America

## Abstract

Insulin-like peptides (ILPs) regulate growth, reproduction, metabolic homeostasis, life span and stress resistance in worms, flies and mammals. A set of insulin producing cells (IPCs) in the *Drosophila* brain that express three ILPs (DILP2, 3 and 5) have been the main focus of interest in hormonal DILP signaling. Little is, however, known about factors that regulate DILP production and release by these IPCs. Here we show that the IPCs express the metabotropic GABA_B_ receptor (GBR), but not the ionotropic GABA_A_ receptor subunit RDL. Diminishing the GBR expression on these cells by targeted RNA interference abbreviates life span, decreases metabolic stress resistance and alters carbohydrate and lipid metabolism at stress, but not growth in *Drosophila*. A direct effect of diminishing GBR on IPCs is an increase in DILP immunofluorescence in these cells, an effect that is accentuated at starvation. Knockdown of irk3, possibly part of a G protein-activated inwardly rectifying K^+^ channel that may link to GBRs, phenocopies GBR knockdown in starvation experiments. Our experiments suggest that the GBR is involved in inhibitory control of DILP production and release in adult flies at metabolic stress and that this receptor mediates a GABA signal from brain interneurons that may convey nutritional signals. This is the first demonstration of a neurotransmitter that inhibits insulin signaling in its regulation of metabolism, stress and life span in an invertebrate brain.

## Introduction

Insulin and insulin-like peptides regulate development, growth, reproduction, metabolism, stress resistance and lifespan in animals from *Caenorhabditis elegans* to mammals [1], [2], [3], [4], [5], [6]. In *Drosophila* there are seven insulin-like peptides (DILP1 - 7), with striking similarities to either insulin, insulin-like growth factor or relaxin [2], [7], [8], [9], [10]. However, only one DILP receptor has so far been identified in *Drosophila* [2], [11]. In adult *Drosophila* DILP signaling plays an important role in metabolic homeostasis, resistance to various stresses and regulation of life span [3], [12], [13], [14], [15]. Three of the DILPs (DILP2, 3 and 5) are produced by a small set of median neurosecretory cells in the *Drosophila* brain and likely to be released into the circulation from neurohemal areas in the corpora cardiaca and anterior aorta [2], [16], [17]. Ablation of the insulin producing cells (IPCs) in the brain results in retarded growth, increased glucose levels in the circulation, increased storage of lipid and carbohydrate of adults, reduced fecundity, and increased stress resistance [12], [17]. This suggests that one or several of the DILPs of the IPCs mediate these responses. Recent experiments where individual DILPs were targeted actually showed that the peptides expressed in the IPCs display redundant functions [10].

It is, however, not clear how the production and release of DILPs are regulated in adult *Drosophila* [1], [4], [17]. Cell autonomous nutrient sensing has so far not been demonstrated for brain IPCs in *Drosophila* [4], [18], [19]. Instead nutritional sensing takes place in adipose tissue, the fat body, that in turn signals to the IPCs (see [4], [18]). A recent paper demonstrated a humoral factor released from the fat body that acts on IPCs, but the chemical identity of this remains to be elucidated [19]. It is likely that there are additional hormonal signals or neuronal inputs that regulate release of DILPs from IPCs, as has been shown for pancreatic β-cells in mammals (see [20], [21], [22], [23]). Hormonal factors have not yet been identified in *Drosophila*, but a brain neuropeptide, short neuropeptide F (sNPF), has been suggested as a stimulator of DILP production in brain IPCs in regulation of larval growth [24]. Another regulator of DILP signaling in the *Drosophila* brain, during development and growth, is serotonin [25]. It is, however, not clear how hormonal factors or neurotransmitters regulate the activity of IPCs in the adult *Drosophila* brain and we set out to identify neuronal pathways that may play such roles.

Analysis of the distribution of metabotropic GABA_B_ receptors (GBRs) revealed expression on brain IPCs, in *Drosophila*, suggesting that GABA is involved in inhibitory regulation of these neurosecretory cells. We therefore undertook an analysis of GABA signaling in relation to IPCs and DILP function. By targeted RNA interference (RNAi) we knocked down GBRs specifically in the IPCs and obtained effects on DILP-levels, life span, stress resistance and metabolism at stress, but not on growth. We found no evidence for expression of ionotropic GABA_A_ receptors on the IPCs and thus GABA mediated regulation of these cells seems to be solely by metabotropic receptors, possibly via inwardly rectifying potassium channels.

## Results

### Insulin producing cells in the brain express GABA_B_ receptors

**Figure 1:**
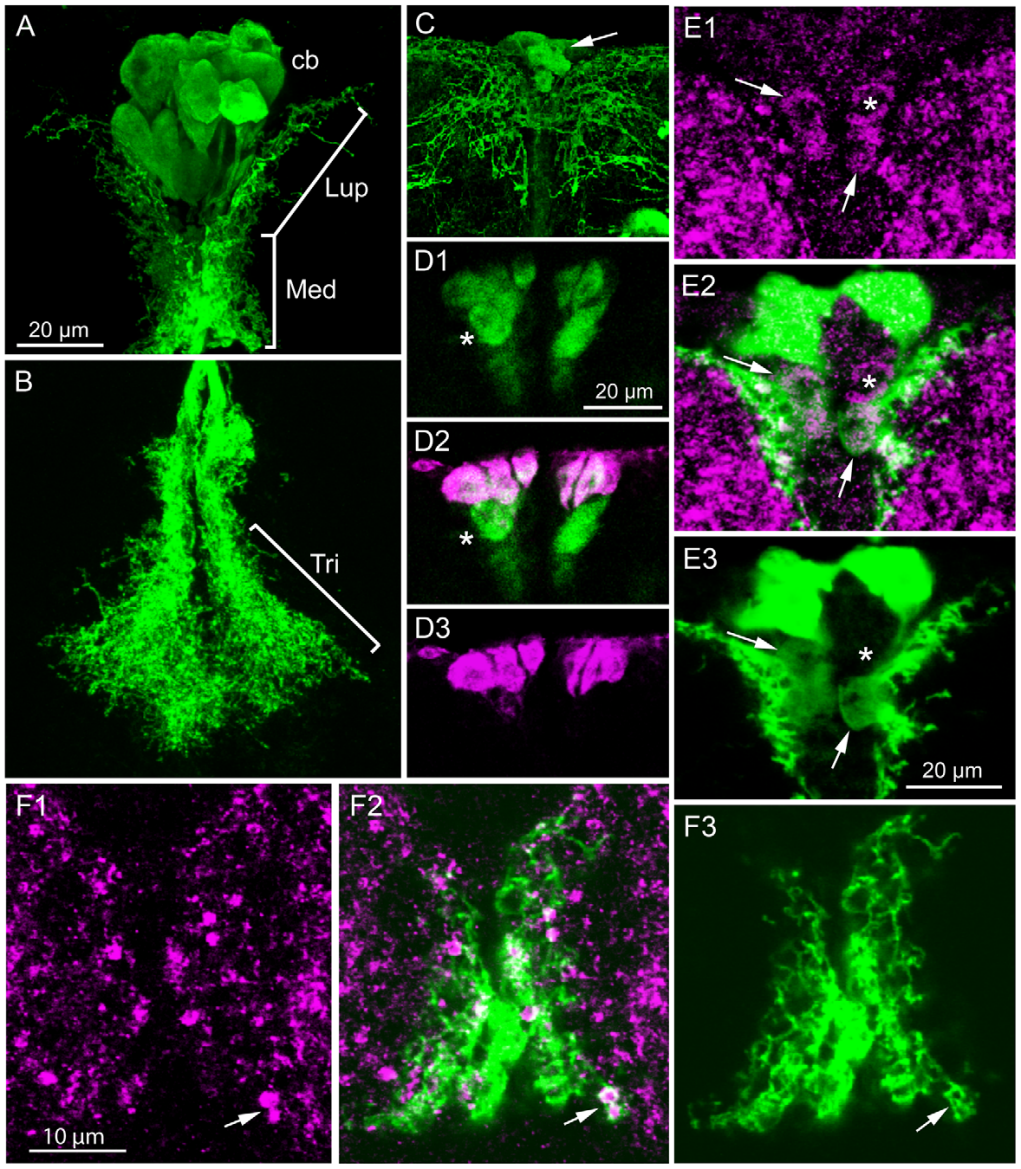
The GABA_B_ receptor (GBR) is expressed on insulin-producing cells (IPCs). **A** and **B**. Dilp2-Gal4-driven GFP in IPCs. The IPCs arborize in three regions: lateral branches in upper pars intercerebralis (Lup), median branches (Med) along median bundle above the central complex and in tritocerebrum (Tri in B). The axons projecting to the corpora cardiaca are not seen in this maximum projection. Cb, cell bodies of IPCs. **C**. GFP driven by GBR2-Gal4 (GABA_B_receptor2) displays the IPCs and additional neurons in the median neurosecretory cell group (arrow). Extensive arborizations can be seen in the pars intercerebralis, a smaller portion of which belong to the IPCs. **D1-3**. The IPCs colocalize DILP2 immunoreactivity (magenta) and GBR2-Gal4 expression (green). A set of MNCs below the IPCs express GBR2, but not DILP2 (asterisk). **E1-3**. Punctate immunolabeling with anti-GBR2 can be seen on the cell bodies of some of the IPCs (e. g. at arrows) and on some of the processes both marked with Dilp2Gal4-GFP (projection of 3 optical sections; for single section see Fig. S1B). One cell body expresses GBR immunolabel, but not Dilp2 (similar to cells marked with asterisk in 1D). **F1-3**. GBR2 immunlabeling of presumed IPC dendrites in the median region above the central complex (region corresponding to Med in 1A).

There is a cluster of median neurosecretory cells in the *Drosophila* brain the produce DILP2, 3 and 5 [2], [16], [17]. These insulin producing cells (IPCs) have arborizations in three regions of the brain: (1) some thin branches extending laterally in dorsal protocerebrum, (2) numerous shorter branches along the IPC neurites in the dorsal part of the median bundle, and (3) extensive arborizations in the tritocerebrum (Fig. 1A, B). The IPC axons terminate in neurohemal areas of the corpora cardiaca and anterior aorta. Here we investigated the expression and functional roles of GABA receptors on the IPCs.

In *Drosophila*, like in mammals, the metabotropic GABA_B_ receptors (GBRs) are G-protein-coupled seven-transmembrane proteins composed of two subunits GABA_B_R1 and GABA_B_R2 [26], [27]. The GABA_B_R1 is the ligand binding unit and GABA_B_R2 is required for translocation to the cell membrane and for stronger coupling to the G-protein [27], [28]. Thus the two subunits are likely to be coexpressed as heterodimers in membranes wherever active GBRs occur. We have used two different markers for the neuronal localization of GABA_B_R2: an antiserum to a part of the GABA_B_R2 protein [29] and a *GABA_B_R2*-Gal4 line [30] to visualize expression with GFP.

Both in larvae and adults the *GABA_B_R2*-Gal4 drives GFP expression in median neurosecretory cells similar in location and morphology to the insulin producing cells (IPCs) in the dorsal protocerebrum (Fig. 1C). To identify which of the GABA_B_R2-expressing neurons that are IPCs we applied antiserum to DILP2. It was clear that most, if not all, the DILP immunolabeled cells also display *GABA_B_R2*-Gal4 expression (Fig. 1D). There are some additional large neurons that express GABA_B_R2, but not DILP immunoreactivity. These are just ventral to the IPCs and likely be other median neurosecretory cells (Fig. 1D).

The antiserum to GABA_B_R2 does not readily label cell bodies in the brain [29], [31], so it can mainly be used for localization of receptor protein at synapses. We applied this antiserum to brains bearing the transgenes *Dilp2*-Gal4;UAS-*GFP*. In these preparations we could detect punctate GABA_B_R2 immunoreactivity on arborizations of the IPCs in the pars intercerebralis and weak immunolabeling of their cell bodies (Fig. 1E, F, Fig. S1A, B). Although GABA_B_R2 immunoreactive punctuates are widespread in the brain, the most prominent localization to IPCs was seen along the short processes of the main neurites (Fig. 1F, Fig. S1). GABA_B_R2 immunolabeling was also seen in cell bodies likely to correspond to the *GABA_B_R2*-Gal4 expressing ones ventral to the IPCs (Fig. 1E, Fig. S1B).

**Figure 2:**
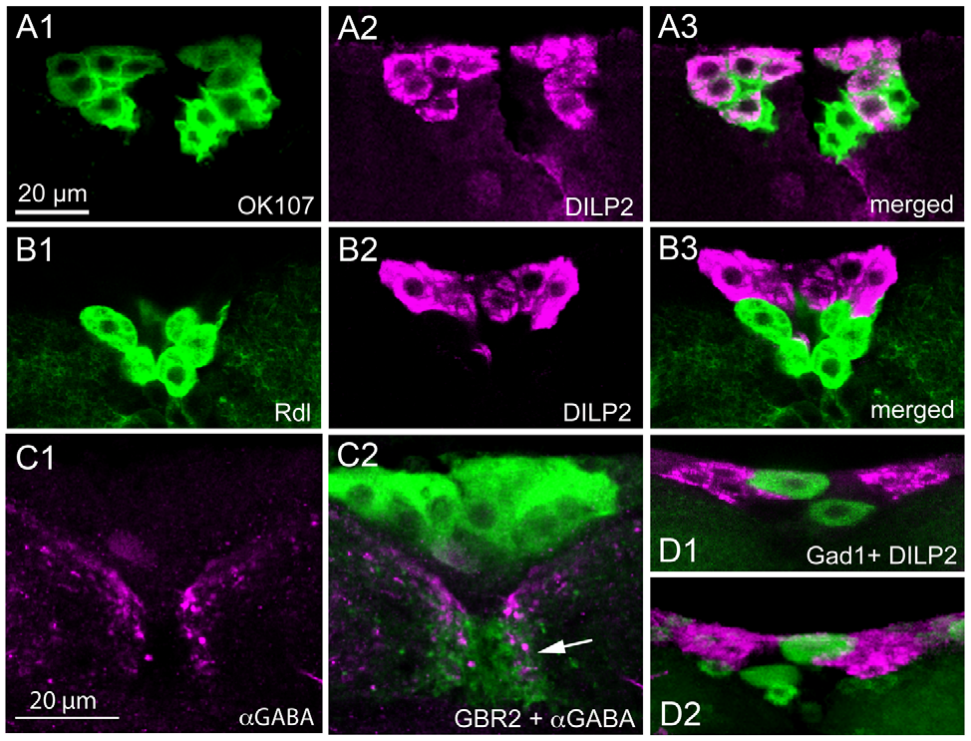
Insulin producing cells express OK107, but not GABA_A_ receptors or GABA. **A1-3**. The enhancer trap Gal4 line OK107 (green) is expressed in the IPCs as seen by DILP2 immunolabeling (magenta). Some additional median neurosecretory cells express OK107, but not DILP2. **B1-3**. The GABA_A_ receptor subunit RDL is visualized here by an rdl-Gal4 driver (green). The rdl-Gal4 expressing cells are not immunolabeled with DILP2 antiserum (magenta). The rdl-positive cells resemble those that express GBR2- and OK107-Gal4, but not DILP2. **C1-2**. Antiserum to GABA (magenta) labels neuronal processes that superimpose (arrow) the IPCs shown in green (GBR2-Gal4-GFP). **D1-2**. The biosynthetic enzyme GAD1 is a good marker for GABAergic neurons. The DILP2 immunolabeled IPCs (magenta) do not express Gad1-Gal4 driven GFP, but several neurons can be seen adjacent to IPCs.

The enhancer trap Gal4 line OK107 has been reported to be expressed in median neurosecretory cells [32], [33]. Here we could show by double labeling with DILP2 antiserum that all the IPCs are included in the OK107 expression pattern (Fig. 2A, Fig. S2C). For some experiments we also employed another Gal4 line known to drive expression exclusively in IPCs, a *Dilp3*-Gal4 [34]. Thus, we have three Gal4 lines that can be used for driving transgenes in IPCs: *Dilp2*-Gal4, *Dilp3*-Gal4 and OK107.

### The GABA_A_ receptor subunit RDL is not expressed on IPCs

Since GABA commonly acts on ion channel receptors, designated GABA_A_ receptors [35], [36], we wanted to determine whether the IPCs also express this type of receptors. The best studied GABA_A_ subunit, RDL (resistance to dieldrin), can form functional homomultimeres [35], and has been mapped to the *Drosophila* brain [31], [37], [38]. For localization of RDL expression we utilized an *rdl*-Gal4 to drive GFP [37]. We found that there is no expression of *rdl*-Gal4-GFP in any of the DILP2 immunolabeled IPCs (Fig. 2B). However, there is *rdl*-Gal4 expression in large cells ventral to the IPCs that may be other median neurosecretory cells, similar to the ventral ones identified by the GABA_B_R2-Gal4 (Fig. 2B). Antiserum to RDL does not label cell bodies at all [31], and requires fixation that precludes GFP visualization, and could therefore not be utilized to support the *rdl*-Gal4 expression.

### GABAergic neurons converge on IPCs

Next we set out to identify the GABAergic inputs to the IPCs. Different markers for GABAergic neurons were used: antisera to GABA and the biosynthetic enzyme GAD1 and two different *Gad1*-gal4 lines. The GABA and GAD1 antisera were applied to flies bearing the transgenes *Dilp2*-Gal4;UAS-*cd8-GFP* or *Dilp3*-Gal4;UAS-*cd8-GFP* and the *gad1*-Gal4-driven GFP was combined with immunolabeling with DILP2 antiserum.

There are large numbers of GABA producing neurons in both the larval and adult brain of *Drosophila* [31], [39]. Thus, it is not trivial to reveal individual neurons with axonal projections that superimpose with IPC branches. It is, however, clear that both GABA and GAD1 immunoreactive neuronal processes arborize in the region of IPC branches in the pars intercerebralis, especially in the areas of presumed IPC dendrites (Fig. 2C). There is clearly no coexpression of the markers for GABA and DILPs in the IPCs, but some cell bodies located adjacent to the IPCs express GABA and GAD1 (Fig. 2D). This means that the IPCs are not GABAergic and, thus, that GBRs on IPCs are postsynaptic. The *Gad1*-Gal4 expression confirms the distribution of putative GABAergic branches adjacent to the IPC dendrites and the lack of coexpression of GAD1 and DILPs in IPCs (Fig. 2D).

### GABA_B_R2 knockdown in insulin producing cells decreases life span of flies

**Figure 3:**
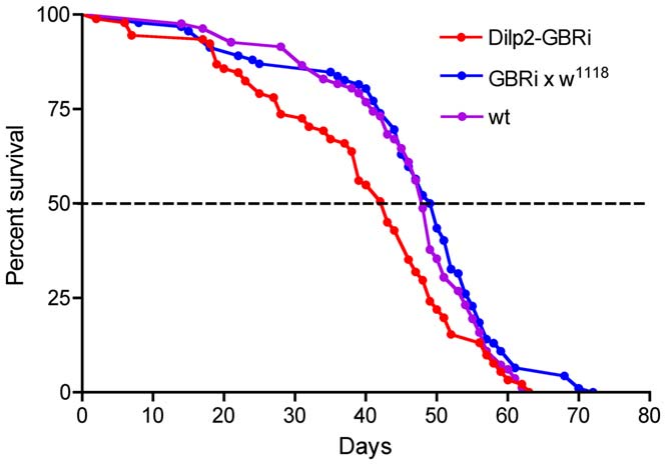
GABA_B_ receptor knockdown on insulin-producing cells diminishes lifespan. Lifespan was determined for normally fed flies with GABA_B_ receptor diminished on IPCs (Dilp2-GBRi) compared to controls (Dilp2-w1118 and wildtype flies, wt). A slight, but significant, reduction of lifespan was seen in the Dilp2-GBRi flies (p<0.001 compared to wildtype flies; p<0.001 compared to GBRi-w1118, Log Rank test; n = 82–91 for the different genotypes).

Insulin signaling influences longevity in *Drosophila* [3], [12], [40]. Thus, as a test of the effect of GABA signaling to IPCs we knocked down the expression of the GABA_B_R2 on these cells and monitored the life span of flies that were fed normally. The efficacy of the UAS-*GBRi* in diminishing GABA_B_R2 levels and GBR function has been described previously [30]. In all experiments in this paper we used male flies, except in a few cases when noted. Flies bearing the transgene *Dilp2*-Gal4;UAS-*GBRi* displayed a slight, but significantly reduced life span compared to both control lines (Fig. 3). This suggests that with diminished GABA signaling the IPCs release more DILPs and as a result the flies display a reduced lifespan. However, the lifespan reduction is not drastic in normally fed flies, suggesting that GABA signaling may primarily inhibit IPC activity under specific conditions (as shown below).

### GABA_B_R2 knockdown in insulin producing cells affects DILP levels

**Figure 4:**
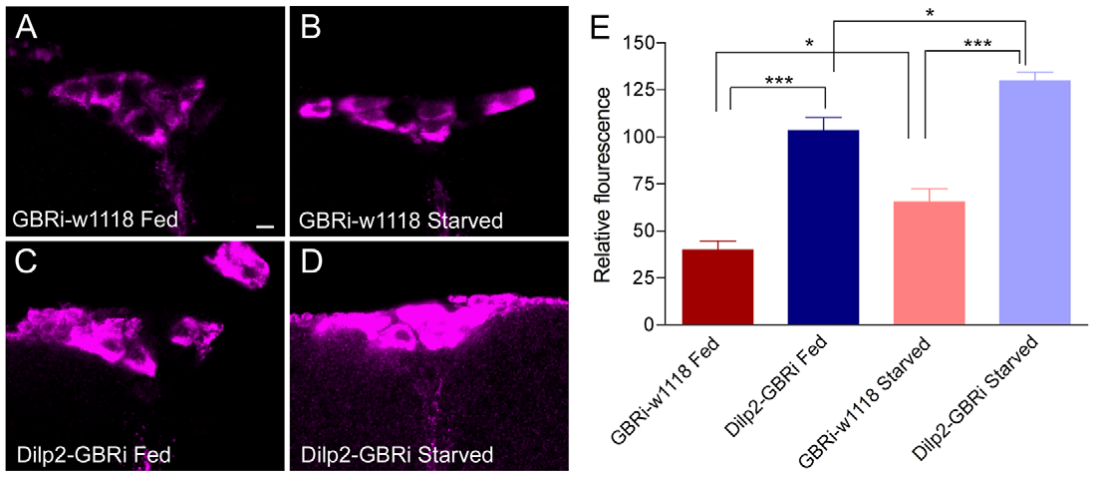
GABA_B_ receptor knockdown on insulin-producing cells affects DILP levels. **A**. Relative DILP immunofluorescence in IPCs in fed and starved flies with and without GABA_B_ receptor knockdown (GBRi) in IPCs (Dilp2-Gal4/UAS-*gbr2*-RNAi). The DILP antiserum used is likely to cross react with DILP2, 3 and 5 [16]. Control flies (GBRi-w1118) display significantly lower levels of DILP-immunofluorescence than the flies with GBRi (Dilp2-GBRi), both in fed flies (p<0.001; Anova with Tukey's comparison) and after starvation (p<0.001). A smaller, but significant, increase in DILP fluorescence is seen at starvation for both genotypes (p<0.05 in both cases). 19–35 cells were measured in 5 specimens of each genotype. **B–E**. representative confocal images of DILP-immunolabeled IPC of the fed and starved control (GBRi-w1118) and experimental (Dilp2-GBRi) flies.

The IPCs in fed adult flies display robust immunolabeling with antiserum to DILP2 (Fig. 4) as shown previously [16], [19]. The antiserum used here, raised against the A-chain of DILP2, is likely to cross react with DILP2, 3 and 5 expressed by the IPCs [16]. We undertook a quantification of the DILP immunofluroescence in the IPCs in fed flies and flies starved for 24 h where GABA_B_R2 expression was knocked down with targeted RNAi by means of the cross *Dilp2*-Gal4/UAS-*GBRi*.

Starved control flies display higher levels of DILP immunoreactivity in IPCs than fed ones, suggesting that during starvation insulin storage is increased as release is reduced (see also [19]) (Fig. 4). Knocking down the GABA_B_R2 in IPCs of fed flies results in an increase in relative DILP-immunofluorescence compared to control flies (Fig. 4), suggesting increased DILP production (or diminished release). In starved flies the GABA_B_R2 knockdown results in a further increase of DILP-fluorescence (Fig. 4). This experiment indicates that the GBR normally inhibits DILP signaling from the IPCs. Since both starved and fed flies display higher DILP levels in cell bodies with reduced GBR expression it seems that GABA signaling affects both DILP production and release; increased release appears to be compensated by increased production in GBR knock-down flies. It can be noted that with a restricted diet the levels of DILPs in the IPCs are differentially affected; DILP5 was decreased whereas DILP2 and 3 were unaffected [15]. Thus, in our experiments we may also affect levels of the three DILPs differentially. The DILP antiserum used here is likely to cross react with all three peptides in the IPCs and therfore the immunolabeling probably reveals the net level of the three DILPs. Our main interest here was, however, to show that manipulating the GBR on IPCs alters DILP levels and future studies will address details on individual peptides.

### GABA_B_R2 knockdown in insulin producing cells decreases stress resistance

Since GBR knockdown affected lifespan of normally fed flies only slightly, we next investigated the role of the receptor in IPCs in the flies' responses to starvation and desiccation. Decreased insulin signaling is known to increase resistance to metabolic stress such as starvation [3], [4], [12], [17], [41]. We therefore investigated the effects on survival at metabolic stress in flies where the GABA_B_R2 was knocked down in IPCs by means of three different Gal4 drivers with expression in these cells.

**Figure 5:**
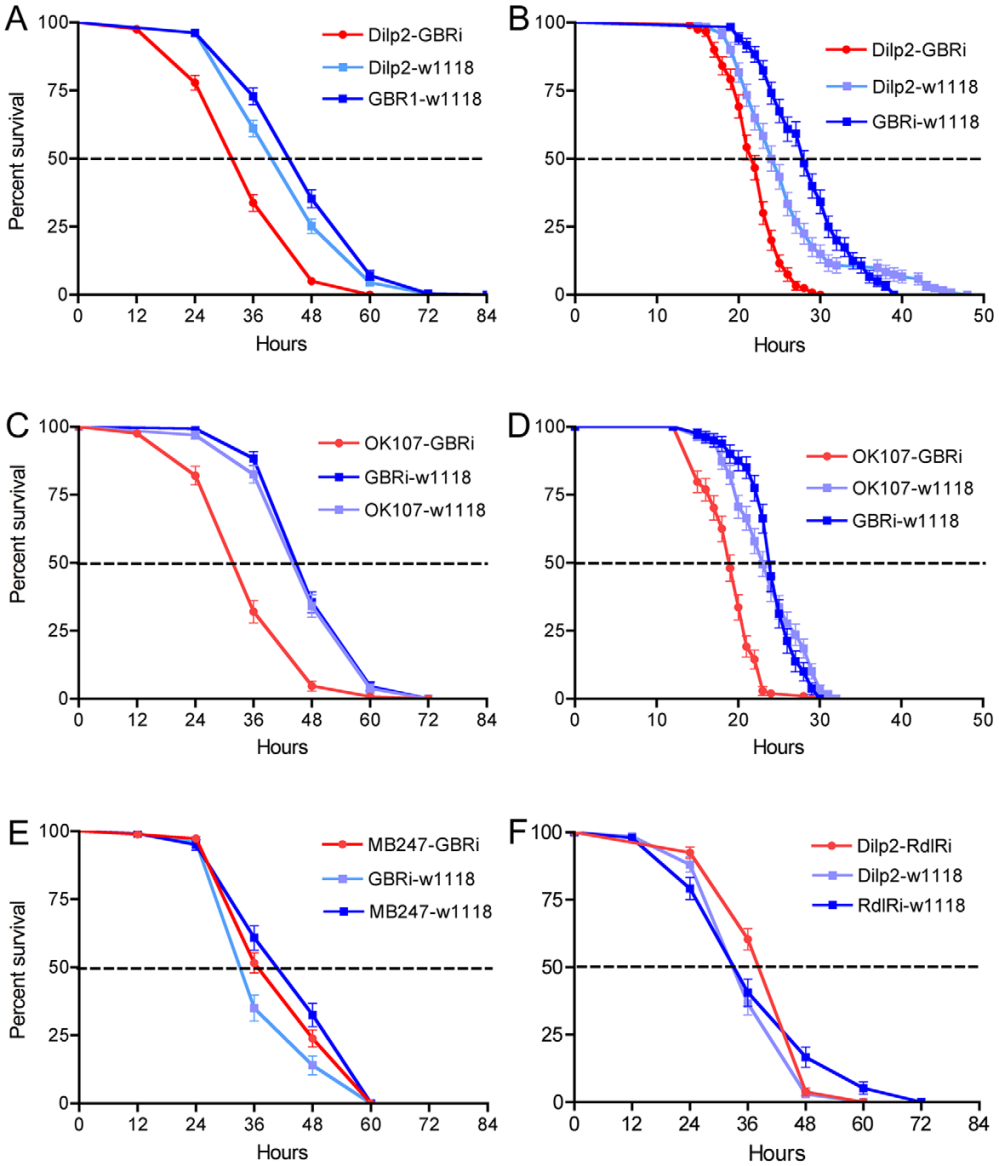
GABA_B_ receptor knockdown on IPCs increases sensitivity to starvation and desiccation. We tested GABA_B_ receptor knock-down with two Gal4 drivers, compared to parental controls, in starvation (flies kept on aqueous agarose) and desiccation (no food and no water). All experiments were run in at least three replicates, unless specified. **A**. Using a Dilp2-Gal4 driver to knock down the GABA_B_ receptor (Dilp2-GBRi) we obtained flies that display significantly reduced survival at starvation (p<0.001, Log rank test; n = 210–270 for each genotype). **B**. The same genotypes were tested for survival during desiccation. (p<0.001; n = 120 for each genotype, 2 replicates). **C**. The OK107 Gal4 driver is also expressed in IPCs and was used for GABA_B_ receptor knock-down (OK107-GBRi). At starvation survival is significantly decreased in OK107-GBRi flies (p<0.001; n = 155–193). **D**. The same genotypes were tested at desiccation. Again, a significant reduction was seen after receptor knockdown (p<0.001; n = 80–120; 2 replicates). **E**. As a control for the OK107 driver, that includes mushroom body Kenyon cells, we utilized a distinct driver for Kenyon cells (MB247) that is not expressed in median neurosecretory cells (IPCs). MB247-driven GBRi does not affect survival at starvation compared to the two parental controls (n = 100–184 for each genotype). **F**. We have no evidence for expression of the GABA_A_ receptor subunit RDL in IPCs. Driving *Rdl*-RNAi in IPCs with the cross Dilp2-RdlRi did not alter survival at starvation. We found no significant difference between the three genotypes (n = 136–181; 2 replicates).

First, two driver lines that seem to be restricted to the IPCs, *Dilp2*- and *Dilp3*-Gal4, were crossed with UAS-*GBRi* flies. GABA_B_R2 knockdown flies that were kept in tubes with aqueous agarose, but no food (starvation), displayed a significantly decreased survival compared to controls (Fig. 5A, Fig. S1A). The *Dilp2*-Gal4 driver was slightly more efficient than the *Dilp3*-Gal4 in producing a strong phenotype at starvation. We also monitored survival in GABA_B_R2 knockdown flies that were exposed to desiccation (neither food nor water). Both Gal4 drivers produced flies that were less resistant to desiccation (Fig. 5B). A second UAS-*GABA_B_R2*-RNAi line (GBRi-V; from VDRC) was crossed to the *Dilp2*-Gal4 flies and tested for survival at starvation. Also this RNAi line induced a significantly abbreviated survival at starvation (Fig. S1C).

We next tested another Gal4 driver, OK107, that includes most, if not all the IPCs, judged by DILP2 immunolabeling (Fig. 2A). The OK107 driven knockdown of the GABA_B_R2 resulted in flies with a strongly reduced survival, both at starvation and desiccation (Fig. 5C, D). Since this driver line also displays strong expression of GFP in the majority of the intrinsic mushroom body Kenyon cells [33], [42], [43] (Fig S1C), we used as a control another driver line, MB247, that displays expression in mushroom body Kenyon cells, but not in IPCs [42]. This is an important control since the Kenyon cells express the GABA_B_R2 (not shown) [31]. Knockdown of the GABA_B_R2 with MB247 did not result in a changed response to starvation (Fig. 5E). These experiments therefore suggest that the GABA_B_R is important in the mediation of the IPC-regulated stress responses and that the Kenyon cells of the mushroom bodies do not contribute to this regulation.

Although we have no evidence for expression of the GABA_A_ receptor subunit RDL in the IPCs (see above), we tested the effect of driving *rdl*-RNAi in these cells. Flies with the transgene *Dilp2*-Gal4;UAS-*rdl*-*RNAi* did not display an altered response to starvation, compared to parental controls (Fig. 5F). Thus it seems that RDL is not utilized by IPCs in control of insulin signaling.

Since the experiments above suggest that the activated GABA_B_R inhibits production and release of DILPs by IPCs and knockdown of the receptor leads to increased DILP signaling, we analyzed the effects of over-expression of DILP2 in the IPCs at starvation. Crossing the transgenes *Dilp2*-Gal4 and UAS-*Dilp2* produced flies that displayed a strongly reduced survival at starvation, similar to that seen after GABA_B_R2 knockdown (Fig. S1D).

Taken together our findings indicate that GABA signaling to the IPCs is more prevalent during metabolic stress, since the reduction of lifespan was more prominent in GBR knock-down flies exposed to starvation or desiccation than in normally fed flies.

### Knockdown of an inwardly rectifying K^+^ channel subunit, possibly associated with GABA_B_Rs, diminishes stress resistance

**Figure 6:**
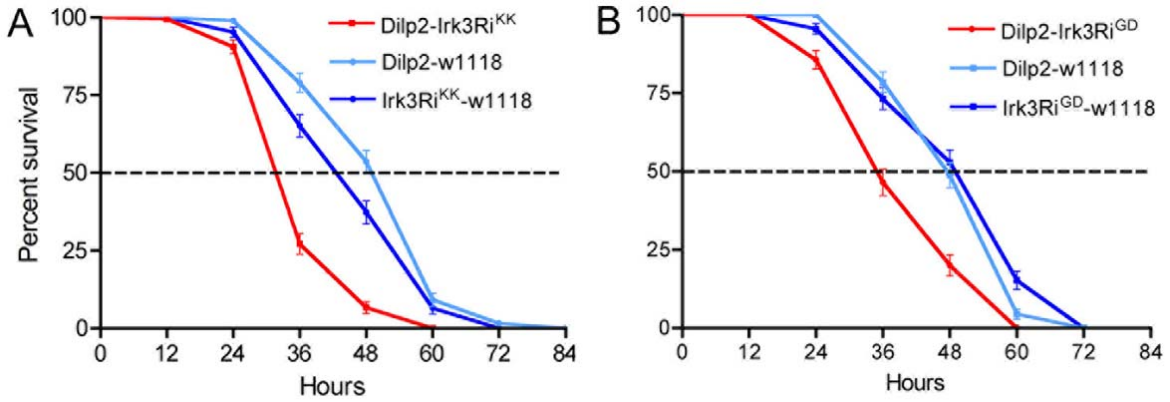
Knockdown of an inward rectifying K-channel mimics GABA_B_ receptor knockdown. Two different UAS-*Irk3*-RNAi lines (Irk3Ri^KK^ and Irk3Ri^GD^) were crossed to Dilp2-Gal4 flies and tested for survival at starvation. Both crosses resulted in flies that survived significantly shorter than parental controls. **A**. Dilp2-Irk3Ri^KK^ flies (p<0.001; n = 169–180 for each genotype; three replicates). **B**. Dilp2-Irk3Ri^GD^ flies (p<0.001; n = 140–158; 3 replicates).

Commonly, postsynaptic GABA_B_Rs, when activated, increase K^+^ conductance through G-protein-coupled inwardly rectifying K^+^ channels (GIRKs) or other K^+^ channels, that induce a hyperpolarization (see [44], [45]). Activation of the *Drosophila* GABA_B_R in heterologous expression systems was shown to increase K^+^ conductance by stimulating GIRKs [26]. To investigate whether elements that may be downstream of the GABA_B_R in IPCs play a role in starvation responses we knocked down a K^+^ channel subunit, Irk3 (CG10369) that may form GIRKs in *Drosophila*. Thus, *Dilp2*-Gal4 flies were crossed with two different UAS-*Irk3*-RNAi lines and exposed to starvation. Both crosses resulted in flies that displayed strongly diminished survival at starvation (Fig. 6A, B), indicating a possible link to GABA_B_R signaling.

### Diminished GABA_B_R signaling in IPCs affects carbohydrate and lipid levels

**Figure 7:**
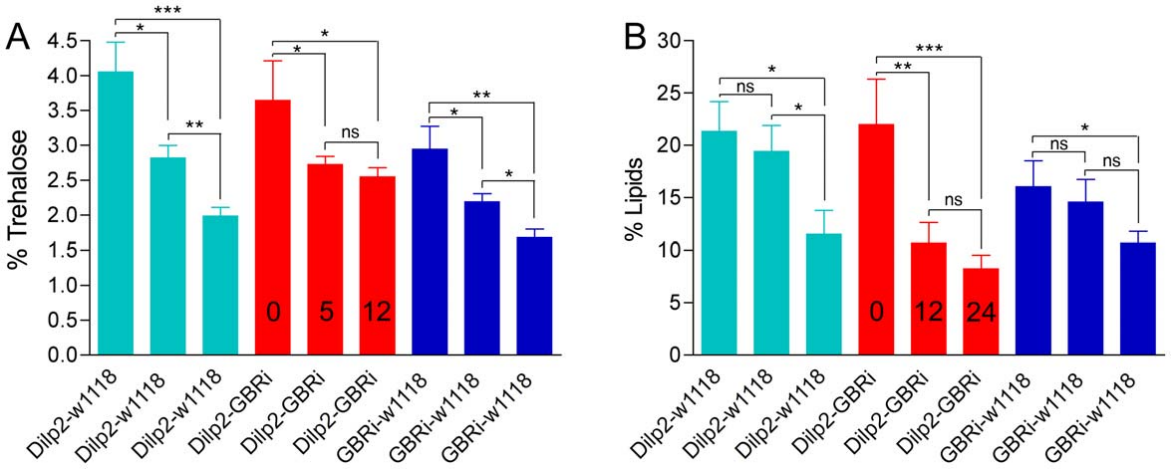
GABA_B_ receptor knockdown affects levels of trehalose and lipid at starvation. **A**. Trehalose levels were determined in fed flies and flies starved for 5 and 12 h (0, 5, 12 in bars) in flies with GABA_B_ receptor knockdown in IPCs (Dilp2-GBRi) and parental controls. In controls the trehalose levels gradually diminish (significantly) after 5 and 12 h starvation, whereas in GBRi flies there is no significant difference between 5 and 12 h starvation (n = 120 for each genotype). Two way Anova [ns, not significant (p>0.05), * p<0.05, ** p<0.01, *** p<0.001]. **B**. Lipid levels were determined in flies of the same genotypes after 0, 12 and 24 h starvation. In controls there is a significantly more drastic decrease in lipids between 12 and 24 h, whereas in GBRi flies there is a much more drastic (and significant) decrease between 0 and 12 h starvation (n = 120 for each genotype). Two-way Anova [ns, not significant (p>0.05), * p<0.05, ** p<0.01, *** p<0.001].

It has been reported that insulin-like peptides regulate stores of carbohydrate and lipid in *Drosophila* [1], [12], [17], [46], [47], [48]. We tested whole body levels of trehalose in *Dilp2*-Gal4/UAS-*GBRi* flies that were fed normally and flies after 5 h or 12 h starvation. All genotypes displayed similar levels of trehalose when fed normally (0 h starvation). In control flies trehalose levels gradually dropped to about 50% after 12 h starvation (Fig. 7A). In flies with GABA_B_R2 knockdown in IPCs this later (5–12 h) drop in trehalose was significantly diminished (Fig. 7A).

Also whole body lipid was measured in normally fed flies and flies exposed to 12 h and 24 h starvation. In control flies lipid levels drop gradually with starvation, especially during the last 12–24 h (Fig. 7B). The flies with GABA_B_R2 knockdown in IPCs, however, display a strongly increased drop in lipid already after the first 12 h (Fig. 7B). Thus, the GABA_B_R2 knockdown flies appear to mobilize lipids at a much higher rate at starvation.

Again, it seems that the role of the GBRs on IPCs is linked to responses to metabolic stress since neither lipid nor trehalose levels were affected in fed GBR knock-down flies compared to controls.

### Interference with metabotropic GABA signaling does not affect growth

Since insulin signaling is known to be important for regulation of growth during development of *Drosophila* [4], [7], [17], [48], [49], we monitored effects of GABA_B_R2 knockdown on the weight of late larvae and adult flies and length of pupae. Larvae bearing the transgenes *Dilp2*-, *Dilp3* or OK107-Gal4 combined with UAS-*GBRi* were kept on normal food and collected either as late third instar larvae, just after transition to wandering stage, as pupae, or as 4–6d old adults. Neither the weight of larvae, nor the size of the pupae with diminished GABA_B_R2 in IPCs differed from parental controls (Fig. S3A, B). The same result was obtained for weights of adult male and female flies (Fig. S3C–F). Thus, in our experiments it appears that diminishing the GABA_B_R on IPCs does not affect growth.

We cannot exclude that the GBR knockdown was less efficient in the larval stages and thus DILP signaling less affected. However, it could also be that the GABA signaling does not affect growth in normally fed larvae, thus supporting the suggestion that the role of GBRs on IPCs may be linked to stress signaling.

## Discussion

We showed here that metabotropic GABA_B_ receptors, GBRs, expressed on insulin producing neurosecretory cells, IPCs, in the *Drosophila* brain are involved in inhibitory regulation of insulin signaling. Hence, knockdown of the essential GBR subunit GABA_B_R2 on IPCs leads to a slightly decreased life span, a strongly decreased resistance to desiccation and starvation and to alterations of carbohydrate and lipid storage during starvation. All these effects are what one would expect to record after increased insulin signaling [1], [3], [4], [12]. We indeed detected an increase in DILP-immunolabeling in IPCs of flies with diminished GABA_B_R2 levels, suggesting increased DILP production. On the other hand, we did not obtain evidence for an effect on growth of normally fed larvae after diminishing the GABA signaling. Thus, it appears that the GABA signaling via GBRs on IPCs primarily mediates effects on metabolism and lifespan at metabolic stress, and that this signaling is part of a stress response. This suggestion is supported by the finding that lifespan of normally fed GBR knock-down flies was less drastically reduced that that of flies that were starved or desiccated.

Earlier reports on DILP signaling in adult *Drosophila* have not directly addressed the signal mechanisms or neuronal pathways responsible for controlling the activity of the IPCs. There are reports that insulin-mediated regulation of growth is under control of short neuropeptide F (sNPF) in the brain during larval feeding stages [24], [50]. The same authors also showed an effect of sNPF on food ingestion in adult flies, however, no evidence was presented that this is linked to insulin signaling. Also serotonergic neurons appear to influence insulin signaling during development and growth [25], although serotonin receptors have not yet been identified on IPCs. Our findings that GABA via its metabotropic receptor affects insulin signaling are therefore the first to demonstrate a direct neuronal inhibitory control of IPCs in the adult fly, and thus the first to show regulation of insulin signaling in metabolism and stress responses, but probably not growth.

In mammals it has been reported that GABA via both the GABA_A_ and GABA_B_ receptors regulate insulin release locally in the pancreas [20], [51], [52]. It was found that activation of GABA receptors in pancreatic beta cells regulates insulin secretion in concert with changing glucose levels; GABA decreases secretory activity in these cells in response to glucose [51], [52]. In *Drosophila* the IPCs express GABA_B_Rs, but not the otherwise abundant ionotropic GABA_A_ receptor subunit RDL. We, however, found other median neurosecretory cells close to the IPCs that can be visualized by a *rdl*-Gal4 line. In support of the lack of RDL expression in IPCs we detected no effect on stress resistance after expression of *rdl*-RNAi with the *Dilp2*-Gal4 driver.

The GABA_B_Rs are known to couple to inwardly rectifying potassium channels, GIRKs [26], [45], [53], [54]. In mammals the GIRKs play an important role in the regulation of neuronal excitation by mediating slow inhibitory synaptic responses and also by contributing to the resting membrane potential [55]. Also in *Drosophila* inward rectifier K^+^ channels of different types have been demonstrated [56], [57] and the *Drosophila* GABA_B_R can couple to GIRKs [26]. We found that knockdown of one of the *Drosophila* inwardly rectifying K^+^ channels Irk3 [57], [58] phenocopies GABA_B_R2-knockdown, possibly suggesting a coupling to the GABA_B_Rs.

Due to massive presence of GABAergic neuron processes in the brain we could not identify the individual GABA expressing neurons that innervates the IPCs. Thus, it is not clear where this GABAergic pathway may receive inputs from nutrient sensing cells or neurons mediating such sensing. It is, however, clear that the IPCs are not GABAergic and therefore the GABA_B_R expression is postsynaptic on these cells. In contrast, certain systems of GABAergic neurons, for instance in the *Drosophila* visual system, can express presynaptic GABA_B_Rs [37].

Our findings that brain IPCs can be inactivated by GABAergic signaling suggests that these cells are under both stimulatory and inhibitory regulation. Production and release of DILPs is induced by a circulating factor released from fatbody [19] and possibly by neuronal sNPF under certain conditions [24], [50] and may be inactivated by GABA, as shown here, and possibly by serotonin [25]. It is not surprising that multiple neuronal systems and hormonal factors regulate the IPCs to fine-tune the production and release of the very important DILPs. Similarly, the insulin release from mammalian beta cells is under control by several neuromediators, such as GABA, serotonin, glucagon-like peptide and other peptides [20], [23], [51], [52], [59], [60], [61]. Also other insect hormones, such as ecdysone and juvenile hormone are under regulation of a number of stimulatory and inhibitory factors (see [62], [63], [64], [65]), indicating that developmental processes and homeostasis requires tight regulatory systems. We therefore expect to detect further modulators of IPC activity in *Drosophila*.

## Materials and Methods

### Fly stocks

For immunocytochemistry we used Oregon R and *w^1118^* strains of *Drosophila melanogaster*, as well as different Gal4 lines crossed with UAS-GFP for expression of green fluorescent protein (GFP). Gal4 lines were also used for driving RNAi constructs in specific sets of neurons. The *GABA_B_R2-*Gal4 (*GBR*-Gal4) and UAS*-GABA_B_R2-RNAi* (UAS-*GBRi*) lines [30] were gifts from Dr. J. W. Wang. To specify insulin-producing cells we used *Dilp2-Gal4* [66], provided by P. Shen, and in a few controls a *Dilp3-Gal4* [34] produced in the lab of M. Pankratz (Bonn, Germany), provided by M. Tatar (Providence, RI). The OK107-Gal4 from Bloomington *Drosophila* Stock Center (at University of Indiana, Bloomington, IN) drives expression in intrinsic neurons of the mushroom bodies [32], [33] and in a cluster of median neurosecretory cells, whereas the MB247-Gal4 [42] provided by R. Tanimoto (MPI Neurobiology, Martinsried, Germany) drives expression almost exclusively in intrinsic neurons of the mushroom bodies (no neurons in pars intercerebralis). This Gal4 strain was crossed with *UAS-dicer2* (w^1118^;P{UAS-*dicer2*,w[+]}) from Vienna *Drosophila* RNAi Center (VDRC, Vienna, Austria) to obtain a stable strain homozygous for *Dicer2* and MB247 to enhance the effect of the RNAi. An *Rdl-*Gal4 (for the GABA_A_ receptor subunit RDL) was a gift from Julie Simpson (HHMI, Janelia Farm, VI) (see [37]). To visualize GABA-producing neurons we used a *Gad1*-Gal4 [67] provided by Dr. G. Miesenböck (Oxford, UK) or another driver for *Gad1*, designated Gad2b-Gal4 [68] from T. Kitamoto (Univ. Iowa, Iowa City, IA). A UAS*-rdl-RNAi* [69] was provided by R. L. Davis (Houston, TX). Another UAS-GABA_B_R2-RNAi (CG6706; UAS-*GBRi-V*) construct and two different UAS-*Irk3*-RNAi lines (CG10369, dKirIII, for putative inwardly rectifying K^+^ channels) were obtained from VDRC [70]. Finally, to over express DILP2 we utilized a UAS-Dilp2 produced in the laboratory of E. Hafen, Zürich, Switzerland [49], [66], obtained from P. Shen (Athens, GA).

### Antibodies

An antiserum to a sequence of the GBR subunit GABA_B_R2, raised in rabbit, was described and characterized previously [29], [31] and used at 1∶16,000. For detection of GABA producing neurons we used rabbit antisera to GABA (Sigma, St. Louis, MO; # A2052) and to GAD1 (glutamic acid decarboxylase 1) obtained from R. Jackson [71] both at the dilution 1∶1000. Rabbit anti-DILP2 (raised against the A-chain) was kindly provided by M. Brown (University of Georgia, USA) and described previously [16]. The working dilution for anti-DILP2 was 1∶1000. To amplify the GFP signal in some specimens we used a mouse monoclonal antibody to GFP (#A-11120; Molecular Probes, Leiden, Netherlands) at 1∶1000.

### Immunocytochemistry

Fly brains were fixed in 4% paraformaldehyde (PFA) in sodium phosphate buffer (PB; pH 7.4). For GAD1 immunostaining, the brains were fixed in Bouin's fixative. Following several rinses in PB, the brains were incubated with primary antibody diluted in 0.01 M phosphate-buffered saline (PBS; pH 7.4), with 0.25% Triton-X and 0.5% bovine serum albumine (BSA) for 48–72 hours. A thorough washing in PBS containing 0.25% Triton-X (PBS-Tx) was followed by incubation in secondary antibody; Cy3-conjugated goat anti-rabbit antiserum or Cy2- or Cy3-conjugated anti-mouse antiserum (Jackson ImmunoResearch, West Grove, PA) at 1∶1500. For cryostat sections fixed heads were immersed in 20% sucrose over night and embedded in Tissue-Tek OCT compound (#4583, Sakura), frozen at −23°C and cut at 20 µm on a cryostat (Leica, CM1850). Specimens were imaged with a Zeiss LSM 510 confocal microscope (Jena, Germany) and processed with Zeiss LSM software and edited for contrast in Adobe Photoshop CS3 version 10.0.1.

### Quantification of immunofluorescence

Immunocytochemistry with DILP2 antiserum was performed on adult brains from starved and fed flies of different genotypes for quantification of immunofluorescence in IPCs. The brains were imaged in a Zeiss LSM 510 confocal microscope with fixed exposure time, using LSM software. The immunofluorescence was quantified in each cell, using Image J 1.40 from NHI, Bethesda, Maryland, USA (http://rsb.info.nih.gov/ij/). The data were analysed with Student's t-test in Prism GraphPad 6.0.

### Assays of longevity and survival during starvation and desiccation

Male flies, 4–6 d old, were used for the different assays. All flies were kept in an incubator with 12∶12 light:dark (LD) conditions, controlled humidity and 25°C. For the longevity test under normal feeding conditions, 20 flies were placed in each plastic container (27×64 mm) with standard *Drosophila* yeast-agar food. A total of at least 80 flies were used for each genotype. Dead flies were counted each day and the remaining flies were flipped into new bottles with fresh food. For the starvation experiments flies were placed individually in 2 ml glass vials with 500 µl 0,5% aqueous agarose. The vials were checked for dead flies every 12 h. In the desiccation assay, individual flies were placed in an empty glass vial and after 12 hours dead flies were counted every hour. These stress experiments were run in three replicates with at least 40 flies of each genotype per replicate.

### Trehalose Assay

Whole body trehalose was measured according to Isabel et al. [72]. In brief, male flies (4–8 days old) of the different genotypes were kept in tubes (5 flies per tube) with food or with 0.5% aqueous agarose for 0, 5 or 12 h. After the experiment starved and fed flies were weighed (wet weight), then incubated for one hour in 500 µl of 70% EtOH. Each tube of flies was sonicated (Sonics and Materials Inc. Danbury CT. USA) for 20 seconds. The samples were centrifuged for 5 min at ×13,200 rpm and 1 ml of the samples and 500 µl of the trehalose standards were placed in 2 ml Eppendorf tubes and dried in a vacuum centrifuge (Savant Speed Vac; Speed Vac Plus Sc110A). To each tube 200 µl of 2% NaOH was added and vortexed. After mixing, samples well, 1.5 ml of fresh Anthrone reagent (Sigma; Cat. #A 1631) was added and vortexed until the sample had a homogenous yellow colour. Samples were then placed in a water bath set at 90°C for 10 minutes. After this incubation period the samples were removed and 100 µl of each sample was placed in a 96 well ELISA plate. Each sample was measured in triplicate on an ELISA plate reader at 620 nm (Labsystems, Multiscan Plus). 40 flies of each genotype was tested in three replicates.

### Lipid measurements

Lipid content was measured in flies of different genotypes after 0, 12 and 24 h of starvation. The lipid content was determined according to the method of Service (1987). Groups of 5 male flies were weighed on a Mettler MT5 Microbalance (Mettler Toledo, Switzerland) to obtain wet weight and subsequently dried at 65°C for 24 h. Flies were then weighed again to obtain dry weight. Lipids were extracted by placing intact dry flies in glass vials containing diethyl ether for 24 h with gentle agitation at room temperature. The diethyl ether was removed and flies were dried for another 24 h and then weighed to obtain lean dry weight. The difference between dry weight and lean dry weight was considered the total lipid content of the flies. 40 flies of each genotype was tested in three replicates.

### Determination of growth rates

As a measure of growth of different genotypes we used the wet weights of larvae and adults and length of pupae. Third instar wandering larvae or 3–6 day old flies were collected and weighed one by one on a Mettler MT5 Microbalance. Adult flies were anesthetized on ice prior to weighing. Each experimental group (genotype) consisted of 40–90 larvae, pupae or flies (details in Figures).

### Graphs and statistics

For statistical analysis and generation of histograms Prism Graphpad 4.0 was used. Log-rank tests (Mantel-Cox) were performed to analyze for trends in survival in the longevity test, and life span during starvation and desiccation. One-way ANOVA's were used to compare weights and lengths of animals. Student's t-test was used to compare the relative fluorescence in IPCs.

## Supporting Information

Figure S1 **A1-3.** Single confocal sections of Dilp2-Gal4 expressing IPCs (green) labeled with antiserum to GABA_B_R2 (magenta). Not punctate immunolabeling of IPC cell bodies (e. g. in circle) and branches on neurites in median bundle (e. g. at arrow and asterisk). **B1-3.** Single confocal sections of Dilp2-Gal4 expressing IPCs (green) labeled with antiserum to GABA_B_R2 (magenta). Some of the IPC cell bodies are more intensely immunolabeled than others (e. g. at arrows). Note also labeling of IPC dendrites (circled). **C.** Expression of OK107 (GFP) in median neurosecretory cells (MNC) and mushroom body intrinsic neurons (MB), seen in stack of confocal mages.
(TIF)

Figure S2 Additional Gal4 driver and UAS-RNAi lines were tested for survival at stress. **A.** A Dilp3-line was crossed to GBRi and flies tested for survival at starvation. GABA_B_ receptor knockdown in IPCs (Dilp3-GBRi) results in significantly reduced survival (p< 0.001; n = 88-155; 2 replicates). **B.** The same fly cross also displays reduced survival at desiccation (p< 0.001; n = 47–60, 1 replicate). **C.** A different UAS-*GBR2*-RNAi line (GBRi-V; from VDRC) was used for knockdown of the GABA_B_ receptor (Dilp2-GBRi-V) in IPCs. A significant reduction in survival is seen compared to controls (p<0.001; n = 168–180 for each genotype; three replicates). **D.** We overexpressed DILP2 in IPCs with the cross *Dilp2*-Gal4/UAS-*Dilp2.* These flies displayed a very strong reduction in survival at starvation compared to parental controls (p<0.001; n = 128–180 for each genotype, three replicates).
(TIF)

Figure S3 Growth is not affected by GABA_B_ receptor knockdown in IPCs. The weight (and length in D) was used to determine whether growth was affected by GABA_B_ receptor knockdown in IPCs. We tested three Gal4 drivers to affect expression in IPCs: *Dilp3*-Gal4, OK107 (A, C, D) and *Dilp2*-Gal4 (B, E, F). **A.** Late feeding third instar larvae of different genotypes were weighed. No significant difference was seen between the genotypes suggesting that laval growth was not affected by receptor knockdown (n = 60–80 for each genotype and sex). **B.** The length of pupae was used to test effects on growth after Dilp2-Gal4-driven knockdown of GABA_B_ receptor. No significant difference in length was detected between genotypes (n = 50 for each genotype; two repicates). **C and D.** A lack of effect on growth was also noted when weighing adult male and female flies of the same genotypes (N = 90 for each genotype and sex). **E and F.** The weights of adult male (E) and female (F) flies using the *Dilp2*-Gal4 driver also revealed no effect on growth after receptor knockdown (n = 185–315 for each genotype and sex).
(TIF)
